# Supplementary material for: Disparities in anti-SARS-CoV-2 reactivity according to vaccines administered in the era of omicron in Cameroon: Lessons for future outbreak response
Source: PLOS Glob Public Health. 2025 Mar 19;5(3):e0004312. doi: 10.1371/journal.pgph.0004312 (PMC11922206; doi:10.1371/journal.pgph.0004312)
Supplement: Attachment — Submitted filename: 31102024 Reviewer.pdf [file pgph.0004312.s001.pdf]

This research on the protection offered by the COVID-19 vaccine is interesting. However, I believe the authors could improve the following points to better engage the reader:

Introduction:
The authors should mention the roles of antibodies in protection and outbreak control.

Materials and Methods:

1. Line 147: The phrase "participants fully complying with the study were finally included" should be clarified. Is this the selection criterion for this research? If so, please provide more details.
2. Line 151: The phrase "systematically screened" needs a more detailed description.
3. Line 190: The reference to the DaAn gene RT PCR kit (www.daangene.com) is incorrect. Please cite the exact kit used in this study.
4. RT-PCR Testing: RT-PCR was used to identify positive cases in this study. However, a small group of participants (4.8% as mentioned in line 244) received the Sinopharm vaccine, an inactivated virus vaccine. Evidence suggests that inactivated virus vaccines can significantly impact RT-PCR results by causing false positives. How do the authors plan to eliminate or identify false-positive RT-PCR results due to contamination from this vaccine?

Results:

1. **Anti-SARS-CoV-2 Antibodies**:
   - Could the authors clarify if anti-SARS-CoV-2 antibodies are higher in the group that received both the infection and the vaccination compared to other groups?
   - In Table 2a, could the authors explain why some groups (9 cases) exhibited symptoms but did not have anti-SARS-CoV-2 antibodies?
   - In Table 2a, could the authors explain why the previously positive group (84 cases) lacked anti-SARS-CoV-2 antibodies? Did these individuals contract the virus naturally? Regarding vaccination and subsequent infection, what should readers understand about the loss of natural antibodies versus vaccine-induced antibodies? Please provide more details.
2. Carefully check the percentage ratios of all the results. Some totals exceed 100%.

Conclusion:
The authors mention that updated vaccines need to address emerging variants. However, it should be clearer and more precise whether new or current vaccines should be used based on the lessons learned from this study.
