# Supplementary material for: Disparities in anti-SARS-CoV-2 reactivity according to vaccines administered in the era of omicron in Cameroon: Lessons for future outbreak response
Source: PLOS Glob Public Health. 2025 Mar 19;5(3):e0004312. doi: 10.1371/journal.pgph.0004312 (PMC11922206; doi:10.1371/journal.pgph.0004312)
Supplement: Attachment — Submitted filename: 271224 Review paper.pdf [file pgph.0004312.s004.pdf]

The authors of "Disparities in anti-SARS-CoV-2 reactivity according to vaccines administered in the era of Omicron in Cameroon: lessons for future outbreak response" demonstrated the effectiveness of different vaccines and their corresponding antibody responses during the Omicron wave. This study also highlights the protection provided by vaccines (approximately 5 months) and emphasizes the need for additional measures to protect the population. However, there are some biased data and unclear information in this research:

1. Why did the authors use two different antibody detection kits, such as Ninonasal and ABBEXA, when the ABBEXA kit covers the Ninonasal kit? How did the authors decide which test kit to use for each sample?

2. The study initially included 2,449 participants, but the authors only analyzed data from 1,987 participants in Table 2a. Could the authors clarify this discrepancy?

3. The authors did not provide the time interval between infection and vaccination. Did they have the vaccination schedule? If so, could they explain why the number of anti-SARS-CoV-2 antibodies decreased after the booster dose (538 vs. 752 in Table 2b)? Should we interpret this as indicating that booster doses are ineffective, or are there other reasons for this decrease? This relates to the conclusion about the effective duration of the vaccine. Do we need booster doses if they are ineffective?
4. The quality of the test kits is crucial for this study. However, the authors did not evaluate the inter-test agreement between the two kits, nor did they assess the rate of false positives compared to RT-PCR. How can the authors ensure the accuracy of their results? If the results are inaccurate, all conclusions will be meaningless. For example, in Table 2a, there were 1,590 cases of individuals with no previous positive results having anti-SARS-CoV-2 antibodies. Conversely, there were 84 cases of individuals without antibodies who had previous positive results. Can this be explained by antibody decay, vaccine ineffectiveness, or test kit quality? These data are unclear to me.
