# Supplementary material for: Disparities in anti-SARS-CoV-2 reactivity according to vaccines administered in the era of omicron in Cameroon: Lessons for future outbreak response
Source: PLOS Glob Public Health. 2025 Mar 19;5(3):e0004312. doi: 10.1371/journal.pgph.0004312 (PMC11922206; doi:10.1371/journal.pgph.0004312)
Supplement: Attachment — Submitted filename: Response to reviewers.docx [file pgph.0004312.s006.docx]

**Responses to Reviewers:**

Journal Requirements:

1. We noticed you have some minor occurrence of overlapping text with the following previous publication(s), which needs to be addressed:

-https://dx.doi.org/10.1128/spectrum.02732-21
-https://doi.org/10.1038/s41579-023-00878-2

In your revision ensure you cite all your sources (including your own works), and quote or rephrase any duplicated text outside the methods section. Further consideration is dependent on these concerns being addressed.

**Response 1:** We thank the managing editor for the comment. However, the said references are already cited in our manuscript (respectively Ref N°16 and Ref N°15).

2. Please review your reference list to ensure that it is complete and correct. If you have cited papers that have been retracted, please include the rationale for doing so in the manuscript text, or remove these references and replace them with relevant current references. Any changes to the reference list should be mentioned in the rebuttal letter that accompanies your revised manuscript. If you need to cite a retracted article, indicate the article’s retracted status in the References list and also include a citation and full reference for the retraction notice.

**Response 2:** We thank the managing editor for the comment. We have gone through the reference list and no amendment was made as all references are still accessible. None have been retracted.

Additional Editor Comments (if provided):

Thank you for submitting your manuscript to PLOS Global Public Health. I am the academic editor working on this manuscript. Before I send it out for peer review, I'd like you to edit your abstract a bit. I have put my comments below. I appreciate your willingness to turn in multiple rounds of manuscript edits before we send this out to peer review, and I think that making the abstract tighter will help me when I look for peer reviewers.

With the advent of COVID-19, anti-SARS-CoV-2 vaccine was a global health priority, but evidence on its significance within tropical settings remained limited. We sought to assess the disparity of anti-Sars-CoV-2 antibodies according to vaccine status and types of vaccines administered in Cameroon during Omicron waves.

- "disparity" here is a bit of a charged word. You could decide to keep it, but disparity typically refers to some difference that results from a historical injustice. I think you just mean "difference"?

**Response 3:** We thank the Academic Editor for the comment. We have rephrased as follows: “… the distribution of anti-SARS-CoV-2 antibodies according to vaccine status…” (see line 47, page 2).

A cross-sectional sero-survey was conducted from February-15 through July-31 2022 among individuals tested for COVID-19 in Yaoundé-Cameroon. Anti-SARS-CoV-2 antibodies (both IgG and IgM) were tested on plasma and statistical analyses were performed with p<0.05 statistically significant.

- generally it's okay to not mention the alpha level in an abstract. instead could you mention things like what kinds of statistical tests you did do? you mention logistic regression later but it's a bit confusing in its context.

**Response 4:** We thank the Academic Editor for the comment. We have removed the part of the alpha level as suggested (see lines 51-52, page 2).

Overall, 2449 participants were enrolled: median-age was 40 [31–49], 56.4% (1382/2449) men, 2.2% (54/2449) with flu-like symptoms and 19.6% (481/2449) reporting previous SARS-CoV-2 positivity. Regarding COVID-19 vaccination, 67.5% (1652/2449) had received at least one dose, 55.0% (909/1652) two-dose series and 37.1% (613/1652) received additional booster doses. Median duration from vaccination to phlebotomy was 5 [4–9] months. Global seroprevalence of anti-SARS-CoV-2 antibodies was 81.1% (1987/2449).

- recommend removing "global" here (could be misconstrued as "world wide")

**Response 5:** We thank the Academic Editor for the comment. We have removed the word as suggested (see line 57, page 2).

Following logistic regressions, vaccine status, booster doses, post-vaccination time (≤5 months), Pfizer and Moderna vaccines, all remained associated with a high prevalence of anti-SARS-CoV-2 antibodies (aOR=1.95, aOR=1.36, aOR=1.64, aOR=2.07 and aOR=1.52 respectively; all p<0.05).

-  the first part of this phrase is confusing, since not sure if "vaccine status" is part of the list with "logistic regression", or if you mean "in a logistic regression, vaccine status (aOR=1.95), booster doses (etc.), ....,  were all associated with a high prevalence of antibodies.

**Response 6:** We thank the Academic Editor for the comment. The sentence was previously written in a contracted form. We have however amended the sentence as suggested (see lines 59-62, page 2).

This high seroprevalence of anti-SARS-CoV-2 antibodies suggests a certain degree of immunity/protection at community-level in Cameroon during Omicron waves, with Pfizer and Moderna inducing greater immunogenicity. However, rapid antibody waning (~5 months) calls for vaccine updates with novel variants (arising from a rapidly evolving virus) that could compromise already acquired immunity.
