# Supplementary material for: Disparities in anti-SARS-CoV-2 reactivity according to vaccines administered in the era of omicron in Cameroon: Lessons for future outbreak response
Source: PLOS Glob Public Health. 2025 Mar 19;5(3):e0004312. doi: 10.1371/journal.pgph.0004312 (PMC11922206; doi:10.1371/journal.pgph.0004312)
Supplement: Attachment — Submitted filename: Response_to_Reviewers_auresp_3.docx [file pgph.0004312.s007.docx]

**Response to Reviewers:**

Journal Requirements:

Please review your reference list to ensure that it is complete and correct. If you have cited papers that have been retracted, please include the rationale for doing so in the manuscript text, or remove these references and replace them with relevant current references. Any changes to the reference list should be mentioned in the rebuttal letter that accompanies your revised manuscript. If you need to cite a retracted article, indicate the article’s retracted status in the References list and also include a citation and full reference for the retraction notice.

**Response**: Reference list is complete and correct.

Additional Editor Comments (if provided):

[Note: HTML markup is below. Please do not edit.]

Reviewers' comments:

Reviewer's Responses to Questions

**Comments to the Author**

1. If the authors have adequately addressed your comments raised in a previous round of review and you feel that this manuscript is now acceptable for publication, you may indicate that here to bypass the “Comments to the Author” section, enter your conflict of interest statement in the “Confidential to Editor” section, and submit your "Accept" recommendation.

Reviewer #4: (No Response)

Reviewer #5: All comments have been addressed

Reviewer #6: All comments have been addressed

Reviewer #7: All comments have been addressed

Reviewer #8: All comments have been addressed

2. Does this manuscript meet PLOS Global Public Health’s [publication criteria](https://journals.plos.org/globalpublichealth/s/criteria-for-publication)? Is the manuscript technically sound, and do the data support the conclusions? The manuscript must describe methodologically and ethically rigorous research with conclusions that are appropriately drawn based on the data presented.

Reviewer #4: Yes

Reviewer #5: Yes

Reviewer #6: Yes

Reviewer #7: Yes

Reviewer #8: Yes

3. Has the statistical analysis been performed appropriately and rigorously?

Reviewer #4: Yes

Reviewer #5: Yes

Reviewer #6: Yes

Reviewer #7: Yes

Reviewer #8: Yes

4. Have the authors made all data underlying the findings in their manuscript fully available (please refer to the Data Availability Statement at the start of the manuscript PDF file)?

The [PLOS Data policy](https://journals.plos.org/globalpublichealth/s/data-availability) requires authors to make all data underlying the findings described in their manuscript fully available without restriction, with rare exception. The data should be provided as part of the manuscript or its supporting information, or deposited to a public repository. For example, in addition to summary statistics, the data points behind means, medians and variance measures should be available. If there are restrictions on publicly sharing data—e.g. participant privacy or use of data from a third party—those must be specified.

Reviewer #4: Yes

Reviewer #5: Yes

Reviewer #6: Yes

Reviewer #7: Yes

Reviewer #8: (No Response)

5. Is the manuscript presented in an intelligible fashion and written in standard English?

PLOS Global Public Health does not copyedit accepted manuscripts, so the language in submitted articles must be clear, correct, and unambiguous. Any typographical or grammatical errors should be corrected at revision, so please note any specific errors here.

Reviewer #4: No

Reviewer #5: Yes

Reviewer #6: Yes

Reviewer #7: (No Response)

Reviewer #8: Yes

6. Review Comments to the Author

Please use the space provided to explain your answers to the questions above. You may also include additional comments for the author, including concerns about dual publication, research ethics, or publication ethics. (Please upload your review as an attachment if it exceeds 20,000 characters)

Reviewer #4: The authors of "Disparities in anti-SARS-CoV-2 reactivity according to vaccines administered in the era of Omicron in Cameroon: lessons for future outbreak response" demonstrated the effectiveness of different vaccines and their corresponding antibody responses during the Omicron wave. This study also highlights the protection provided by vaccines (approximately 5 months) and emphasizes the need for additional measures to protect the population. However, there are some biased data and unclear information in this research:

1. Why did the authors use two different antibody detection kits, such as Ninonasal and ABBEXA, when the ABBEXA kit covers the Ninonasal kit? How did the authors decide which test kit to use for each sample?

**Response**: We thank the reviewer for this comment. As stated in the Introduction section (lines 125-129), these were the test kits available locally. They were all delivered by the Ministry of Public Health to all health facilities performing the analysis nationwide.

2. The study initially included 2,449 participants, but the authors only analyzed data from 1,987 participants in Table 2a. Could the authors clarify this discrepancy?

**Response**: We thank the reviewer for this comment. However, statistical analyses were effectively performed on the entire population (i.e. 2449). “1987” represents the number of people with anti-SARS-CoV-2 antibodies in the entire population. We can’t relate with your point.

3. The authors did not provide the time interval between infection and vaccination. Did they have the vaccination schedule? If so, could they explain why the number of anti-SARS-CoV-2 antibodies decreased after the booster dose (538 vs. 752 in Table 2b)? Should we interpret this as indicating that booster doses are ineffective, or are there other reasons for this decrease? This relates to the conclusion about the effective duration of the vaccine. Do we need booster doses if they are ineffective?

**Response**: We thank the reviewer for these observations. Time between infection and vaccination was not analyzed because nearly all participants with previous infection merely recalled the exact date they were infected. There was no vaccination calendar in the country; participants willingly adhere to vaccination. Finally, there were relatively more people with anti-SARS-CoV-2 antibodies in the group of those with booster doses than in the group of those with two-dose series [538/(538+75) vs. 752/(752+157) i.e. 87.76% vs 82.73]; we cannot therefore say that booster doses were ineffective. It’s a matter of proportion of the populations that have been enrolled.

4. The quality of the test kits is crucial for this study. However, the authors did not evaluate the inter-test agreement between the two kits, nor did they assess the rate of false positives compared to RT-PCR. How can the authors ensure the accuracy of their results? If the results are inaccurate, all conclusions will be meaningless. For example, in Table 2a, there were 1,590 cases of individuals with no previous positive results having anti-SARS-CoV-2 antibodies. Conversely, there were 84 cases of individuals without antibodies who had previous positive results. Can this be explained by antibody decay, vaccine ineffectiveness, or test kit quality? These data are unclear to me.

**Response**: We thank the reviewer for this comment. As mentioned above, we could not evaluate the inter-test agreement as the kits were been delivered at different timepoints by the health authorities. Also, given the cross-sectional design we employed, the observation on previous positivity and antibodies detection cannot be interpreted separately from other findings. We however agree that several reasons could help understand this specific observation including also “time between previous infection and vaccination” or even “the recall bias” due to the fact that participants were the ones reporting previous infection themselves and some of them may have had flu-like symptoms without specifically confirming SARS-CoV-2 infection.

Reviewer #5: • Congratulations to the research team for the clarity of the objectives of the study and the strong statistical analysis conducted.

• I will suggest reorganizing the abstract to have a clear structure/format: Background, methodology, findings and conclusion for clarity and readability.

**Response**: We thank the reviewer for this suggestion. We have reorganized the abstract as recommended.

• I was unclear about the significance of the specific antibody types, IgG and IgM, and their relevance and whether the presence of IgG, IgM, or both was of any public health significance to the study results or their interpretation?

**Response**: We thank the reviewer for this comment. We did not insist on the significance of specific antibody types because IgM were very very low in the population (1.2%).

• Lines 233/234: Table 1, change the percentage on pregnant women from 1,2 to 1.2

**Response**: We thank the reviewer for this comment. We have amended accordingly in the table.

• In the discussion line 384 and 385 made it clear that the study did not assess the titer of antibodies resulting from vaccination but rather evaluated the anti-SARS-CoV-2 reactivity through rapid antibody tests. Therefore, in the conclusion, the phrase in line 402, 403 and 404 ‘’ Interestingly, vaccination with Pfizer and Moderna (i.e. mRNA vaccines) led to higher levels of anti-SARS-CoV-2 antibodies compared to other vaccines type’’ appear confusing. Please rephrase for clarity indicating Pfizer and Moderna vaccines being associated with greater immunogenicity. Do ensure that it does not contradict the statement in the limitation of the study.

**Response**: We thank the reviewer for this comment. We had already stated that in the conclusion (see line 406).

Reviewer #6: 1. In the abstract section the methodology part needs little more elaboration and the results part can be made short by omitting the irrelevant sentences.

**Response**: We thank the reviewer for this comment. We have revised accordingly.

2. The background is written properly.

**Response**: We thank the reviewer for this comment.

3. The methodology is written properly in details regarding the laboratory procedure. However, the statistical analysis part is not properly written. These tests should be performed by any statistical software, but the authors have mentioned that they have done these tests by MS Excel. How? Please explain.

**Response**: We thank the reviewer for this comment. However, analyses were not done with MS Excel but rather Excel sheets served for data entry. We confirm that statistical analyses were performed by statistical softwares (Epi Info and SPSS).

4. The Abs were measured by Lateral flow immunoassay. Do the authors evaluate the validity of this test? Please explain. Is these are the validated kits to detect the Abs?

**Response**: We thank the reviewer for this comment. Yes, the kits were validated for antibodies detection (see references N° 22 and 23).

5. Why PCR was performed on 3rd day? Please explain.

**Response**: We can’t relate with this observation. We did not mention that anywhere in the manuscript.

Reviewer #7: The manuscript genuinely provided information about the anti-SARSCOV-2 antibodies reactivity across all available kinds of COVID-19 vaccines administered during the era of Omicron in Cameroon. It is original and highlights the most potent and immunogenic vaccines administered during this era. I would like to recommend the manuscript for publication in the next PLOS Global Health Edition.

**Response**: We thank the reviewer for this comment/recommendation.

Reviewer #8: Dear authors, thank you very much for drafting this very important informative manuscript. I personally have a few minor comments for authors.

1. The abstract does not indicate the method used especially related to analysis.

**Response**: We thank the reviewer for this comment. We have revised accordingly.

2. Given the nature of the study design and the levels of anti-SARS-CoV-2 antibodies for each vaccine, I would like to see one of the recommendations being to further investigate each vaccine especially the Pfizer vaccine.

**Response**: We thank the reviewer for this comment. We have added the recommendation as suggested (see lines 406-407).

3. What are some of the potential biases that readers should consider when interpreting the findings of this study? I would like to see one of the findings looking at enrollment site

**Response**: We thank the reviewer for this comment. Study limitations are stated in the discussion section (see lines 383-396).

7. PLOS authors have the option to publish the peer review history of their article ([what does this mean?](https://journals.plos.org/globalpublichealth/s/editorial-and-peer-review-process#loc-peer-review-history)). If published, this will include your full peer review and any attached files.


**Do you want your identity to be public for this peer review?** If you choose “no”, your identity will remain anonymous but your review may still be made public.


For information about this choice, including consent withdrawal, please see our [Privacy Policy](https://www.plos.org/privacy-policy).

Reviewer #4: **Yes:** Diep The Tai

Reviewer #5: No

Reviewer #6: **Yes:** Dr. Md. Abdullah Yusuf

Reviewer #7: No

Reviewer #8: No

[NOTE: If reviewer comments were submitted as an attachment file, they will be attached to this email and accessible via the submission site. Please log into your account, locate the manuscript record, and check for the action link "View Attachments". If this link does not appear, there are no attachment files.]

While revising your submission, please upload your figure files to the Preflight Analysis and Conversion Engine (PACE) digital diagnostic tool, https://pacev2.apexcovantage.com/. PACE helps ensure that figures meet PLOS requirements. To use PACE, you must first register as a user. Registration is free. Then, login and navigate to the UPLOAD tab, where you will find detailed instructions on how to use the tool. If you encounter any issues or have any questions when using PACE, please email PLOS at figures@plos.org. Please note that Supporting Information files do not need this step.
